# Effect of *Withania somnifera* on Sleep-Wake Cycle in Sleep-Disturbed Rats: Possible GABAergic Mechanism

**DOI:** 10.4103/0250-474X.49130

**Published:** 2008

**Authors:** A. Kumar, H. Kalonia

**Affiliations:** University Institute of Pharmaceutical Science, Panjab University, Chandigarh-160 014, India

**Keywords:** Sleep, sleep deprivation, EEG, EMG

## Abstract

Sleep deprivation disrupts significantly sleep pattern and cause poor quality of sleep. The aim the present study was to explore role of *Withania somniferra* root extract in sleep-disturbed rats. Male wistar rats (n=5-6/group) were sleep deprived for 24 h using grid suspended over water method. *Withania somniferra* extract (100 mg/kg) was administered intraperitoneally (i.p.) 30 min before actual recording (EEG and EMG) recording and electrophysiological recordings are further classified as- sleep latency, slow wave sleep, paradoxical sleep, total sleep, wakefulness. One day (24 h) sleep deprivation delayed latency sleep, reduced duration of slow wave sleep, rapid eye movement sleep, total sleep time and increased total waking as compared to animals placed on saw dust (P<0.05). Pretreatment with *Withania somniferra* extract (100 mg/kg) and diazepam (0.5 mg/kg) significantly improved electrophysiological parameters, which was further reversed by picrotoxin (2 mg/kg) and potentiated by muscimol (0.05 mg/kg). Flumazenil (2 mg/kg) did not produce any significant effect on the sleep parameters of *Withania somnifera* root extract. Present study suggests the involvement of GABAergic mechanism in the sleep promoting effect of *Withania somniferra* in sleep-disturbed state.

Insomnia (i.e. difficulty in falling asleep) and other sleep disorder such as RLS^1^ are common complaints among the middle-aged populations in different countries. Sleep is defined as a combined impression from the electrophysiological parameters (EEG: electroencehalogram, EMG: electromyogram and EOG: Electro occulogram). Polysomnogram helps to identify, analyze the stages of sleep^2^ and to evaluate the sleep pattern of hypnotic drugs. Sleep deprivation has been reported to change the normal sleep pattern significantly^3^ and benzodiazepines increase hypnotic effects of drugs (non-REM sleep time) in rats placed on the grid^3^–^4^. There have been some reports comparing the characteristics of hypnotic activities of drugs on the sleep wakefulness cycle in animals^5^–^6^. However, most of these studies were done using normal animals, therefore making it difficult to evaluate accurately the pharmacological effects of hypnotics. New sleep disturbance model in rats is useful for estimating the characteristics of some hypnotics. It seems likely that this model was subjected to a relatively powerful stress in the insomnia model developed in the previous study because the animals were exposed to two stressors (grid and water). Many hypnotics showing different durations of effects have been developed and widely used clinically. Benzodiazepines are widely used to manage the sleep related problems and have been reported to act by GABAergic system^7^–^8^. The role of GABA in the regulation of sleep has already been well-documented^5^. *Withania* root extract and its constituents have been reported to have mild to moderate hypnotic action and antioxidant activity^9^–^11^. But no study yet reported the effect of *Withania* root extract in sleep-disturbed animals. Therefore, the objective of the present study was to investigate the effect of *Withania somnifera* root extract and their possible interactions with GABAergic modulators on the sleep-wake cycle in sleep-disturbed rats (i.e. grid suspended over water method).

Male Wistar rats (250-300 g) bred in Central Animal House, Panjab University, Chandigarh. The animals were kept under standard laboratory conditions, maintained on 12-h light/dark cycle and had free access to food and water. Animals were acclimatized to laboratory conditions before the test. Each animal was used once in the experiments. All the experiments were performed between 9.00 and 17.00 h. The experimental protocols were approved by Institutional Animal Ethics Committee and were conducted according to the Indian National Science Academy Guidelines for the use and care of experimental animals. Electrodes for polygraphic recording of the electroencephalogram (EEG) and electromyogram (EMG) were implanted in rats as described in Paxinos and Watson^12^. Rats were anaesthetized with pentobarbitone sodium (45 mg/kg, i.p.). Two stainless steel electrodes were implanted over the right parieto-occipital cortex with the help of rat sterotaxic control and an epidural grounding electrode was placed over the frontal cortex. To record EMG, two silver wire electrodes were inserted bilaterally into the dorsal neck muscles. The leads from all the electrodes were then fixed to the skull with dental cement and two additional screws were inserted in the skull to aid fixation of the connector to the skull. Before suturing, an antibiotic ointment (gentamicin) was applied to the incision to prevent infection. Following surgery, rats were housed individually and allowed at least one week to recover before being used for experiment. Animals were kept separately for habituation in the recording chamber two days before actual recording. The rat was placed in its home cage where it could move freely to some extent on the day of experiment. Recording were done at 9.00 am onward. Recording was started 30 min after drug administration and lasted for four hours. Animals were sleep deprived by placing on the grid floor (29×15×7cm) suspended over water (1 cm above) for 24 h as described by *Shinomiya*^3^,^4^. The stainless steel rods of the grid were 3 mm wide and set 2 cm apart from each other. Food and water were provided *ad libitum.* Recording was made by a four-channel polyrite recorder (Recorder and Medicare, Chandigarh) using chart speed of 25 mm/s. The half-amplitude frequency was set at 1-35 for the EEG and 30-75 for four-channel physiological recorder at a chart speed of 25 mm/sec made the EMG. EEG waves were classified^13^ as, sleep latency (SL), slow wave sleep (SWS), paradoxical sleep (PS), total sleep (TS), wakefulness (W). A low-voltage EEG and active EMG showed the animal was being awaked (W), high voltage EEG which composed to slow waves indicated slow wave sleep (S), and low voltage EEG with EMG disappeared remaining small EEG spike was characteristics of paradoxical sleep (P). The total time of SWS and PS was defined as total sleep time (TS). In order to minimize the stress involved in experimental procedure, all rats were habituated to a separate recording chamber and recording conditions. One rat was tested at a time and all rats in a specific drug group were tested once in the experiment. Animals were divided into eleven groups. Naive and control (sleep deprived) were treated as group 1 and 2. Diazepam (0.5 mg/kg, i.p.), *Withania* root extract (100 mg/kg, i.p), flumazenil (2 mg/kg, i.p), and picrotoxin (2 mg/kg, i.p.), muscimol (0.05 mg/kg, i.p.) treated as group 3-7, respectively. Combinations of *Withania* root extract (100 mg/kg, i.p) with diazepam (0.5 mg/kg i.p.), or picrotoxin (0.5 mg/kg, i.p), or muscimol (0.05 mg/kg, i.p.) and or flumazenil (2 mg/kg, i.p.) were treated as group 8-11, respectively. Picrotoxin, flumazenil was dissolved in a few drops of dimethylsulfoxide (DMSO) and then made-up with water. One specific group of animals was assigned to a specific drug treatment each group consist of five to six (n=5-6) animals. All the values are expressed as mean±SEM. The data was analyzed using analysis of variance (ANOVA) followed by Turkey test. In all the tests, criterion for statistical significance was *P*<0.05.

Twenty four hour of sleep deprivation delayed sleep latency, reduced duration of slow wave sleep, REM sleep, total sleep time and increased total waking as compared to animals placed on saw dust (P<0.05). Pretreatment with *Withania* root extract (100 mg/kg) and diazepam (0.5 mg/kg) significantly shortened sleep latency, increased duration of slow wave sleep, total sleep time and decrease total waking as compared to control (sleep-disturbed rat) (P<0.05). However, combination of *Withania* root extract (100 mg/kg) with diazepam (0.5 mg/kg) did not cause further improvement in the sleep promoting effect of *Withania* root extract (Table 1). Flumazenil (2 mg/kg) *per se* did not produce any significant effect on sleep-wake activity of a sleep-disturbed animal. Further, combination of flumazenil (2 mg/kg) with *Withania* root extract (100 mg/kg) did not have any significant effect on sleep-wake activity as compared to their effect *per se*. Picrotoxin (2 mg/kg), a GABA antagonist, produced a convulsive state (head and body twitching, spasmodic activity) in most of animals. Picrotoxin significantly delayed sleep latency, reduced total sleep time and increased waking as compared to control (sleep-deprived animals). Further, combination of picrotoxin (2 mg/kg) with *Withania* root extract (100 mg/kg) significantly reversed hypnotic activity of *Withania* root extract in sleep-deprived animals (P<0.05). Muscimol (0.05 mg/kg), a GABA agonist, signficantly reduced latency to sleep and increased slow wave sleep, REM, total sleep time and decreased total waking as compared to control (sleep-deprived). Combination of muscimol (0.05 mg/kg) with *Withania* root extract (100 mg/kg) significantly potentiated the hypnotic activity of *Withania* root extract as compared to their effect *per se* in sleep-deprived animals (Table 1).

**TABLE 1:** EFFECTS OF *WITHANIA* ROOT EXTRACT AND ITS INTERACTION WITH GABAERGIC MODULATORS ON SLEEP-WAKE CYCLE OF SLEEP-DISTURBED RATS

Drug treatment (mg/kg)	Sleep Latency (Mean±SEM)	Slow Wave Sleep (Mean±SEM)	REM (Mean±SEM)	Total sleep time (Mean±SEM)	Total Waking (Mean±SEM)
Naïve	12.8±2.8	140.4±4.8	10.9±1.6	151.3±5.9	88.7±5.8
Control	28.1±2.2^a^	88.2±5.7^a^	4.8±0.8^a^	93.0±5.9^a^	147.0±4.8^a^
Ash (100)	15.4±2.2^b^	160.7±5.9^b^	3.0±1.2^b^	163.7±5.2^b^	76.3±5.2^b^
Dia (0.5)	8.0±2.0^b^	163.1±4.9^b^	3.2±0.4^b^	166.3±4.6^b^	73.7±4.7^b^
Flu (2)	15.0±21	135.4±3.5	7.0±1.3	142.4±3.5	97.5±3.6
PTX (2)	240.0±0.0	0.0±0.0	0.0±0.0	0.0±0.0	240.0±0.0
Mus (0.05)	10.0±2.1^b^	180.0±3.1^b^	3.8±2.9^b^	183.8 ± 4.0^b^	56.2±4.0^b^
Ash (100)+ Dia (0.5)	8.4±3.6^ns^	174.3±7.7^ns^	2.5±0.6^ns^	176.8±7.4^ns^	63.2±7.4^ns^
Ash (100)+ Flu (2)	17.2±3.7^b^	170.2±4.5^b^	3.0± 4.2	173.2±4.3^b^	66.8±4.5^b^
Ash (100) +PTX (2)	38.1±4.0^b^,^c^	76.4±6.7^b^,^c^	4.4±1.1^b^,^c^	80.8±7.4^b^,^c^	159.2±6.8^b^,^c^
Ash (100)+Mus (0.05)	5.0±2.0^b^,^d^	220.0±3.0^b^,^d^	2.1±3.5^b^,^d^	222.1±2.9^b^,^d^	17.9±2.8^b^,^d^

Values are expressed Mean±SEM.

a *P*<0.05 as compared to naïve (without sleep deprived).

b *P*<0.05 as compared to control (sleep-deprived).

c *P*<0.05 as compared to PTX (2)

d *P*<0.05 as compared to Mus (0.05)

ns Not Significant (ANOVA followed by Tukey test)

Disturbed sleep is one the commonest encounters of day-to-day life in the fast moving society^14^,^15^ and requiring appropriate diagnosis and management. Benzodiazepines are among the most commonly consumed drugs worldwide for the management of sleep and related problems. These drugs facilitate sleep throughout the night and no residual action by the following morning. Most of these studies have been performed on normal animals. Despite recent advances in the development of newer hypnotics in modern medicine, a significant proportion of patients with insomnia, both locally and internationally consume herbal hypnotic regularly^16^. Therefore, there is always a search for a candidate molecule for the better management of sleep. *Withania somnifera* is widely known for its antioxidant, weak hypnotic effect^9^ and reported to act by GABAergic modulation^17^,^18^. In the present study, we have used sleep disturbance model that can be best used to evaluate of hypnotic properties of drugs. Besides, new sleep disturbance model is also useful for estimating sleep pattern alterations or changes due to sleep deprivation. Animals suspended over grid for 24 h significantly altered sleep-wake cycle (delayed sleep latency, shortening of slow wave, REM sleep and total sleep time and increased waking) in rats as compared to animals placed on saw dust. Sleep deprivation through grid has been reported to cause oxidative stress in animals^2^,^3^. Pretreatment with *Withania somnifera* shortened sleep latency, decreased waking, increased NREM and total sleep time in sleep-disturbed rats. These indicated that *Withania somnifera* may have their role in sleep promotion in sleep-disturbed states and can be employed as drugs for the management of sleep and related problems. Diazepam that augments the action of GABA at the GABA_A_ receptor, shortened sleep latency; decreased waking, increased NREM and total sleep time significantly as compared to control (sleep-deprived animals). The above observations again reconfirm the hypnotic action of diazepam in sleep-disturbed state. In the present experiment, diazepam, when administered in combination with *Withania* root extract did not improve sleep promotion of *Withania* root extract in sleep-disturbed animals.

It is well known that GABA_A_ receptor is a supra-molecular complex together with a central-type BZD, comprising several recognition sties (such as BZD, picrotoxin and GABA sites)^19^. To determine the involvement GABA_A_ receptor mechanism, present study explored the combinatorial effects of *Withania* root extract with several GABAergic modulators such as flumazenil, picrotoxin and muscimol. Flumazenil is a specific antagonist of the BZD ligand site on the GABA_A_ receptor. In the present study, combination of flumazenil (2 mg/kg) with *Withania* root extract (100 mg/kg) did not produce any significant influence on the sleep wake cycle of sleep-disturbed rat. This suggests that *Withania* root extract may not bind to BZD site to produce their hypnotic effect in a sleep-disturbed state. Muscimol is a selective GABA_A_ agonist and picrotoxin, GABA antagonist^20^. In the present study, picrotoxin reversed and muscimol potentiated the hypnotic effect of *Withania* root extract in sleep-disturbed rats. The above observations indicate that sleep-promoting activity of *Withania* root extract is linked to allosteric modulation of some of the components of the GABA_A_ receptor supramolecular complex in brain and might be bound to the picrotoxin site on the GABA_A_ receptor. These recognition sites may therefore be another target for the hypnotic action of *Withania* root extract. These also indicate that GABAergic system is involved in the sleep promoting effect of *Withania* root extract in sleep disturbed state.

In summary, the present study showed that *Withania* root extract induced sleep-promoting effect by involving GABAergic modulation, which was significantly antagonized and potentiated by picrotoxin and muscimol, respectively.
